# Signatures of mRNA Alternative Polyadenylation in *Arabidopsis* Leaf Development

**DOI:** 10.3389/fgene.2022.863253

**Published:** 2022-04-26

**Authors:** Zhibo Yu, Liwei Hong, Qingshun Q. Li

**Affiliations:** ^1^ Key Laboratory of the Ministry of Education for Coastal and Wetland Ecosystem, College of the Environment and Ecology, Xiamen University, Xiamen, China; ^2^ Graduate College of Biomedical Sciences, Western University of Health Sciences, Pomona, CA, United States

**Keywords:** alternative polyadenylation, leaf development, Arabidopsis, 3′-end formation, co-transcriptional regulation

## Abstract

Alternative polyadenylation (APA) of pre-mRNA is an important co-transcriptional mechanism that modulates gene expression, leading to transcriptomic and functional diversities. The role of APA in *Arabidopsis* leaf development, however, remains elusive. We applied a poly(A)-tag sequencing (PAT-seq) technique to characterize APA-mediated regulation events in cotyledon and in five stages of true leaf development. Over 60% APA was identified in genes expressed in leaves, consistent with the results in previous publications. However, a reduced APA level was detected in younger leaves, reaching 44% in the 18th true leaf. Importantly, we also found that >70% of the poly(A) site usages were altered in the second true leaf relative to the cotyledon. Compared with the cotyledon, more genes in the second true leaf tended to use the distal site of 3′UTR, but this was not found in pairwise comparison among other true leaves. In addition, a significant APA gene was found to be decreased in a pairwise comparison among true leaves, including differentially expressed genes. The APA genes identified herein were associated with specific biological processes, including metabolic and cellular processes and response to stimuli and hormones. These results provide a new insight into the regulation of *Arabidopsis* leaf development through APA.

## Introduction

Leaves are the main plant organ responsible for photosynthesis through light energy harvesting and driving circulation through transpiration. Leaf morphogenesis in eudicots is divided into four stages. First, the founder cells are recruited from the peripheral zone of the shoot apical meristem (SAM) at the site of the incipient leaf primordium to undergo extensive cell division. Second, the growth occurs along the adaxial–abaxial, proximal–distal, and medio–lateral axes. Third, the leaf blade and petiole regions are established. Finally, across the entire blade, cell proliferation and expansion occur, which leads to both distal and lateral leaf expansion (Du et al., 2018). In this dynamic and precise process, extensive coordination and regulation of gene activities are crucial, which requires the whole program of gene expression circuitry, including mRNA processing control, e.g., pre-mRNA splicing and polyadenylation.

Indeed, the leaf development is controlled by a complex mechanism (Bar and Ori, 2014). During leaf initiation, the expression of *Shoot Meristemless* (*STM*) is required for maintaining the fate of the SAM cells in *Arabidopsis* (Long et al., 1996). Once a leaf primordium has been initiated, a peg-like structure grows and proliferates from the flank of the SAM along its adaxial–abaxial, proximal–distal, and medio–lateral axes to establish leaf polarity. In *Arabidopsis thaliana*, members of the class III homeodomain-leucine zipper (HD-ZIP III) family, including revoluta (REV), phabulosa, and phavoluta, specify the cell identity on the adaxial surface of the leaf (Manuela and Xu, 2020). In fact, HD-ZIP III genes are regulated by miR165 and miR166 (Zhu et al., 2011). In addition, the auxin response transcription factors ARF3 and ARF4 play a key role in adaxial/abaxial polarity (Sarojam et al., 2010). Then, growth along the proximal–distal axis determines the length of the leaf. The genes *Blade-On-Petiole1* (*BOP1*) and *BOP2* belong to the BTB family, which regulates the proximal–distal patterning of the leaf by inducing *AS2* and repressing *KNOXI* (Ha et al., 2003; Jun et al., 2010). Finally, the growth along the medio-lateral axis determines the width of the leaf. Pressed flower/wuschel-like homeobox3 (PRS/WOX3) and WOX1, the two most important regulators, promote medio–lateral growth (Nakata et al., 2012). Interestingly, *WOX1* and *PRS* genes are activated by the HD-ZIP III genes. The expression of the HD-ZIP III gene *REV* overlaps with the expression of *PRS* (Guan et al., 2019). Therefore, the steps in the establishment of leaf polarity may overlap with each other.

Following initiation and polarity establishment, the leaf primordium begins to expand until it has acquired its final shape and size. The molecular machinery underlying this progression balances cell proliferation with cell expansion. Auxin regulates both the AP2-domain transcription factor aintegumenta and CYCD3; 1 to influence both cell proliferation and cell expansion (Mizukami and Fischer, 2000). The deficiency of ARF2 promotes cell proliferation and leads to an increase in the leaf size (Okushima et al., 2005). In addition, gibberellin and brassinosteroids (BR) also promote the growth of the leaf blade because the deficient mutants of these genes show small leaves, whereas the overexpression of these genes produces larger than normal leaves (Mitchum et al., 2006; Oh et al., 2011; Zhiponova et al., 2013). As abovementioned, although it is well known that leaf development is modulated by transcription factors, hormones, miRNAs, and so on, the mechanism of leaf ontogeny remains to be elucidated. The overall process of leaf ontogeny proceeds along a fairly long timescale, but the leaf morphology is predictable. The continuous process of typical dicot leaf ontogeny and morphogenesis has been monitored using anatomical markers (Poethig, 1997). Furthermore, analyses of transcriptome dynamics during leaf development have identified multiple expression patterns of gradual increase, decrease, or a single expression peak with age. From the analyses of leaf development transcriptome, a quantitative definition of the dynamic leaf was established, termed as a digital differentiation index, to predict the leaf age (Efroni et al., 2008). However, the role of co-transcriptional regulation in leaf development has not been reported.

Polyadenylation is an important co-transcriptional processing step, which is essential for the maturation of eukaryotic precursor mRNAs (pre-mRNAs) before they are exported from the nucleus to the cytoplasm (Elkon et al., 2013). This process is a two-step reaction, cleavage of the nascent mRNAs, and addition of a poly(A) tail, which requires the recognition of the poly(A) signal located in the pre-mRNA sequence with a multi-protein complex, and regulates mRNA stability, nuclear export, and translation (Thomas et al., 2012). Expressed sequence tags and high-throughput studies have found that over 70% of genes in mammals, yeast, and plants harbor multiple cleavages and polyadenylation sites (poly(A) sites), and this phenomenon is termed as “alternative polyadenylation” (APA) (Shen et al., 2008; Wu et al., 2011; Derti et al., 2012). Poly(A) sites can be located at different regions of a gene, which may impact the length of the encoded protein or alter the presence of the regulatory elements in the 3′UTR to modulate mRNA stability. Thus, APA enriches the complexity and diversity of transcriptome and proteome and plays a key role in the regulation of gene expression (Xing and Li, 2011). Accumulating evidence has demonstrated that APA is involved in numerous biological processes and it is also subject to many regulations, including the epigenetic process (Lin et al., 2020). In humans, APA is related to disease occurrence, immunity regulation, cancer formation, and cell reprogramming (Mayr and Bartel, 2009; Carpenter et al., 2014). In plants, APA functions in flowering time regulation, seed dormancy, root development, and stress response (Zhang et al., 2008; Bruggeman et al., 2014; Zhang et al., 2015; Cyrek et al., 2016; Lin et al., 2017). The role of APA in leaf development has not yet been reported.

In this study, we applied a poly(A) tag sequencing (PAT-seq) approach to study dynamic APA during *Arabidopsis* leaf development. We found that the ratio of APA genes shows a downward trend in the progression of leaf development, suggesting that APA might be involved in this process. In addition, the number of significant APA genes and DE genes was found to decrease in the true leaf comparison. GO terms of significant APA genes were related to metabolic processes, cellular processes, and response to a stimulus. In addition, during true leaf development, there is a tendency that more genes use proximal sites in 3′UTR. Our results show that genome-wide APA mediates *Arabidopsis* leaf development and contributes to the molecular mechanism of the regulatory process of gene expression.

## Materials and Methods

### Plant Growth Conditions

*Arabidopsis thaliana* ecotype Columbia (Col-0, ABRC stock # CS60000) plants were grown on soil in a growth chamber condition under a light/dark cycle of 12/12 h at 22°C. Then cotyledon and different true leaves, including the second true leaf (2 TL), 6, 10, 14, and 18TL, were collected containing three biological replicates (each repeat contained ten leaves, each leaf from an individual plant) in 5-week-old plants, and the leaves were placed in liquid nitrogen and stored at −80°C.

### RNA Extraction and PAT-Seq Library Construction

Total RNA extraction was performed using the TRIzol reagent (Invitrogen). Following isolation, the DNA in RNA samples was removed using DNase I (Takara) and was purified in an RNA clean column, and then 2 µg of the total RNA was used to construct PAT-seq libraries as described previously (Yu et al., 2019). Briefly, the total RNA was fragmented at 94°C for 4 min, followed by poly(A) enrichment with oligo (dT)_25_ magnetic beads (New England Biolabs). Next, the fragments of poly(A) enriched RNA was reverse transcribed using barcoded oligo (dT) primers with the SMARTScribe enzyme (Clontech) for 2 h, and then 5’ adapter for template switching was added after 2 h. The cDNAs were purified using AMPURE XP beads (Beckman), and then were amplified with PCR for 18 cycles with Phire II (Thermo Fisher Scientific). The PCR product was run on a 2% agarose gel, and 300- to 500 bp fragments were purified to produce PAT-seq libraries. Qualified and quantified libraries were checked by Agilent Bioanalyzer 2100, Qubit 2.0, and qPCR.

### High-Throughput Sequencing and Data Processing

The libraries were sequenced on the Illumina HiSeq 2500 platform at the facility located in the College of the Environment and Ecology, Xiamen University. The sequencing data were processed as described previously (Wu et al., 2011; Yu et al., 2019). Briefly, the low-quality reads, barcodes, and poly(T) stretch of raw reads were filtered out and trimmed. Then valid poly(A) reads were mapped to the *Arabidopsis* reference genome using Bowtie2 software. Next, a list of poly(A) clusters (PACs) was generated, where poly(A) sites (PAs) within 24 nucleotides were grouped into one poly(A) cluster (PAC). For the subsequent analyses, total reads of PACs among all samples with <10 were discarded to avoid uncertainty from low read counts.

### Poly(A) Site Usage Analysis

Poly(A) site usage (PAU) represents the ratio of reads in one PAC relative to a gene total reads (Ha et al., 2018). The average of PAU among three biological replicates were used for calculating cumulative distribution function (CDF) and plotted using the mountain plot package in R. The details of the methods were described previously (Yu et al., 2019). Then genes passing through the following filtering criteria were considered as APA switching events: 1) total read count >10 for a gene; 2) absolute value (PAU of one age leaf–PAU of another age leaf)≥0.1; 3) at least one PAC belongs to significant differences PAC.

The 3′UTRs of TAIR9 genome annotation were extended by 120 nt. For those protein-coding genes without an annotated 3′UTR, their 3′UTRs lengths were defined as 338 nt, which is the average 3′UTR length 218 nt in *Arabidopsis* plus 120 nt extended lengths after the annotated stop codons, as defined previously (Wu et al., 2011). For 3′UTR APA analyses, the average weighted length of each 3′UTR of a gene was calculated as described previously (Fu et al., 2016). The 3′UTR length of the proximal poly(A) site was defined as a1 nt (the distance from the poly(A) site to the start of the 3′UTR), PAT number is b1; the 3′UTR length of distal poly(A) site was defined as a2 nt, PAT number is b2. Then, the average weighted length of 3′UTR of each gene was (a1*b1+a2*b2)/(b1+b2). A cut-off *p value* of 0.01 was used to justify significantly longer or shorter 3′UTR.

### Genome-Wide DE-PACs, DE Genes, and GO Analysis

Differential expression PACs (DE-PAC) were identified using the DESeq2 package. The criterion of a statistically significant difference was an adjusted *p* value < 0.05. All PATs of a gene were summed for representing gene expression levels. Similarly, DE genes were analyzed using the DESeq2 package and an adjusted *p* value < 0.05 threshold was considered to mark significant differences. GO analyses of specific DE-PAC APA genes among different comparison groups were carried out using agriGO (v.2.0), singular enrichment analyses (SEA) was chosen and the *Arabidopsis* Information Resource 10th annotation acted as the background. False discovery rate (FDR) corrected *p* value < 0.05 were selected as statistically significant.

## Results

### Dynamic APA in *Arabidopsis* Leaf Development

**FIGURE 1:**
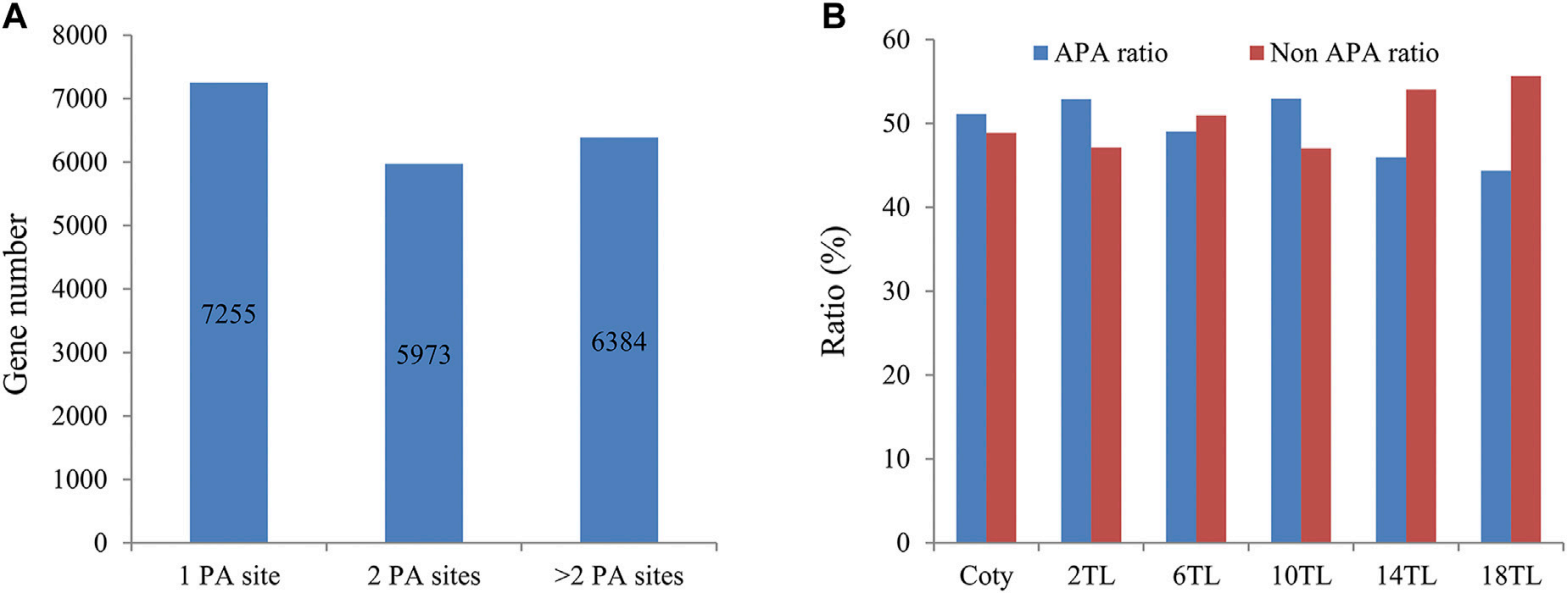
Analysis of APA gene numbers and ratios. **(A)** Numbers of genes with different counts of poly(A) sites. PA site, Poly(A) site. **(B)** Ratio of APA and non-APA genes in each leaf stage. Cotyledon, coty; true leaf, TL; number before TL indicates the order of the leaves, for example, 2TL, the second true leaf.

We set out to understand the involvement of genome-wide APA during *Arabidopsis* leaf development. To this end, we employed PAT-seq to profile dynamic polyadenylation across cotyledon (abbreviated as Coty) and different true leaf (or TL) according to leaf development sequence, including the second true leaf (2 TL), the sixth true leaf (6 TL), Supplementary Figure S1 gives an overall view of the status of each leaf in a pool of wild-type plants at 5-week post-germination. The principal component analysis indicated that three independent repetitions showed good similarity (Supplementary Figure S2), indicating that the sequence data were reliable. Then, a summary of the raw reads, mapped poly(A) tag reads (PATs), and poly(A) site clusters (PACs) for each library are presented in Table 1, suggesting that sufficient data were obtained for further analyses. The distribution of PACs and PATs at the different genic regions was analyzed (Supplementary Figure S3). Altogether, 60% of PACs and over 80% of PATs fell within the annotated 3′UTRs. However, no significant difference was seen in terms of PAT and PAC distribution between different leaves. In total, 56,620 PACs were obtained, as shown in Supplementary Data 1. These PACs mapped to 19,612 genes (69% of total genes in *Arabidopsis*). Of these, the percentage of more than one PAC (defined as APA genes) is 63% (12,357/19,612) (Figure 1A), which is similar to previous research (Wu et al., 2011). Interestingly, we found that the ratio of APA genes shows a downward trend during different leaf development, in particular, it drops to 44% in the 18 TL (Figure 1B), suggesting that APA is related to different leaf development.

**TABLE 1:** Summary of PAT mapping results.

—	Raw reads	Low-quality reads	Aligned 0 times	Aligned 1 time	Aligned >1 times	Mapping rate (%) (≥1 times)	Mapping reads (≥1 times)
coty1	13,110,749	166	4,916,641	6,829,234	1,208,288	62.05	8,037,522
coty2	10,406,926	111	4,166,763	4,919,695	1,185,421	59.44	6,105,116
coty3	10,806,857	117	3,675,884	5,840,682	1,186,974	65.66	7,027,656
2TL1	13,921,895	204	4,628,089	6,863,257	1,844,644	65.30	8,707,901
2TL2	13,829,188	254	4,487,013	7,067,870	1,708,453	66.17	8,776,323
2TL3	14,909,321	211	5,121,198	7,055,336	2,019,471	63.93	9,074,807
6TL1	13,357,605	181	3,974,659	7,149,646	1,983,966	69.68	9,133,612
6TL2	9,832,647	138	2,956,663	4,867,155	1,802,079	69.28	6,669,234
6TL3	13,612,258	235	3,802,441	7,678,274	1,874,983	71.53	9,553,257
10TL1	15,637,872	156	5,287,350	8,159,397	8,159,397	64.81	9,737,565
10TL2	16,358,810	184	5,351,825	9,023,224	1,748,208	66.81	10,771,432
10TL3	20,240,823	228	6,978,634	10,512,931	1,864,415	63.95	12,377,346
14TL1	10,708,077	157	3,513,101	5,364,295	1,648,874	66.63	7,013,169
14TL2	10,903,631	172	3,670,794	5,388,690	1,653,462	65.73	7,042,152
14TL3	11,604,919	182	3,849,704	6,108,380	1,453,748	66.27	7,562,128
18TL1	7,285,299	96	2,917,136	3,310,416	955,538	59.39	4,265,954
18TL2	10,098,462	184	3,314,204	5,234,603	1,422,401	66.76	6,657,004
18TL3	11,769,020	204	3,872,856	5,712,802	2,039,622	66.69	7,752,424

Refer to Supplementary Figure S1 for leaf number annotation. PAT No., numbers of mapped reads [to remove low-quality reads, invalid poly(T) reads, and unmapped tags]; PAC No., numbers of PACs obtained after grouping poly(A) sites that lie within 24 nucleotides of adjacent sites; rep, repeat.

**FIGURE 2:**
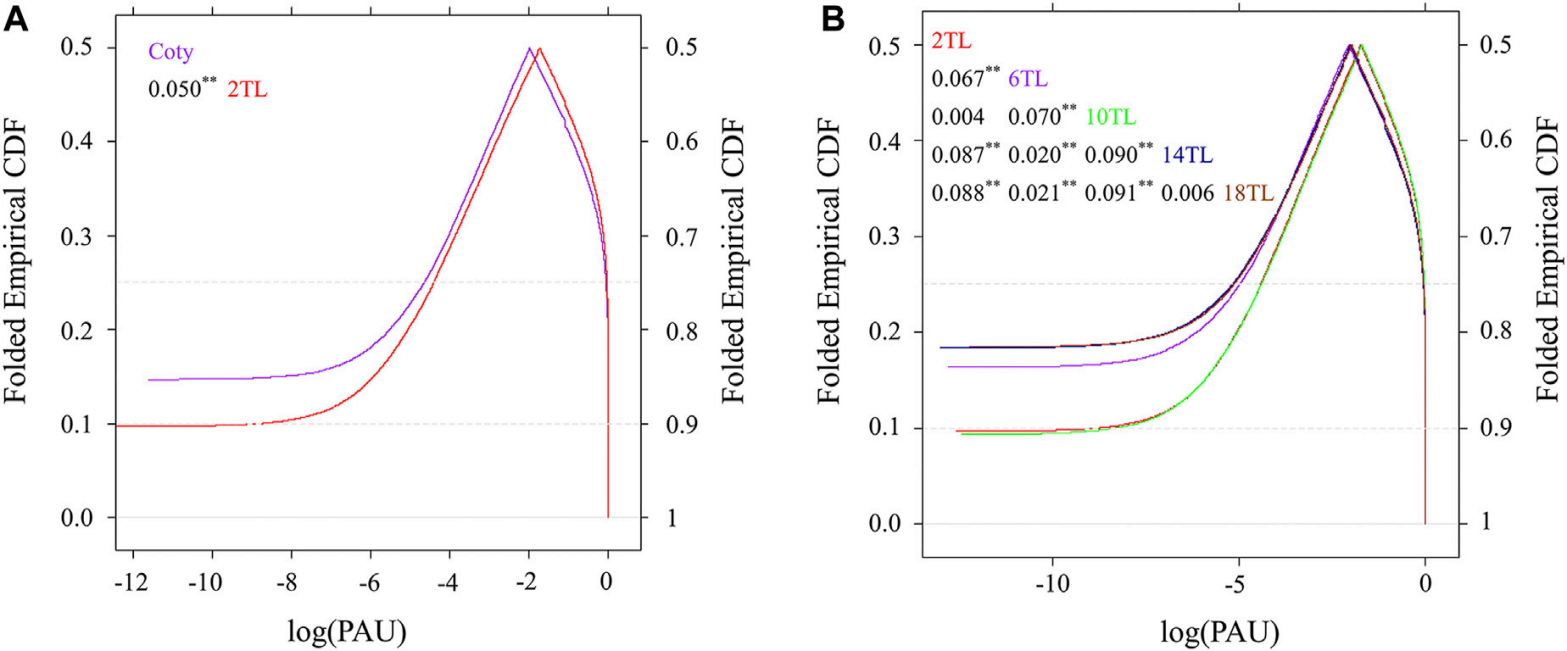
Curve of cumulative distribution function (CDF) based on poly(A) site usage (PAU). PAU values were calculated as the ratio of its expression in all isoforms of a gene. The *x*-axis shows the log PAU based on the average of three biological replicates. The *y*-axis shows folded empirical CDF. The curve of CDF was based on a mountain plot to examine PAU distribution. The Kolmogorov–Smirnov (K–S) test was used to detect the differences in both location and shape of the empirical CDF between different samples. The numbers in the figure insert represent the maximum distance in paired comparison between two CDF curves. **p value < 0.01. **(A)** Comparison of cotyledon and the second true leaf. **(B)** Comparison between true leaves. Leaf annotations are the same as those in Supplementary Figure S1.

To further illustrate the role of APA in leaf development, the relative abundance of each isoform within one gene was calculated and referred to as poly(A) site usage (PAU). PAU values were plotted using a cumulative distribution function (CDF) at the genic region level (Figure 2). Compared to cotyledon, 70% PAU of 2 TL was altered, because CDF curves merge 0.7 on the right *y*-axis (Figure 2A). In addition, the start site of CDF and the median (the point where the curves are folded) differed among 2 TL and cotyledon, indicating unique poly(A) site was used between these two samples. During true leaf development, the maximum distance of adjacent leaf comparison of CDF had a gradually increased tendency (Figure 2B) indicating that APA might play an important role in mediating leaf development. However, the CDF curves almost coincided except for slight differences at the start and end sites. These could be due to having a similar leaf shape (14 TL and 18 TL), or no overall difference in the cumulative fashion (2 TL and 10 TL). Nevertheless, there seem to have three different patterns among these comparisons (Figure 2B).

### Identification of PACs and Genes Displaying Differential Usages

**FIGURE 3:**
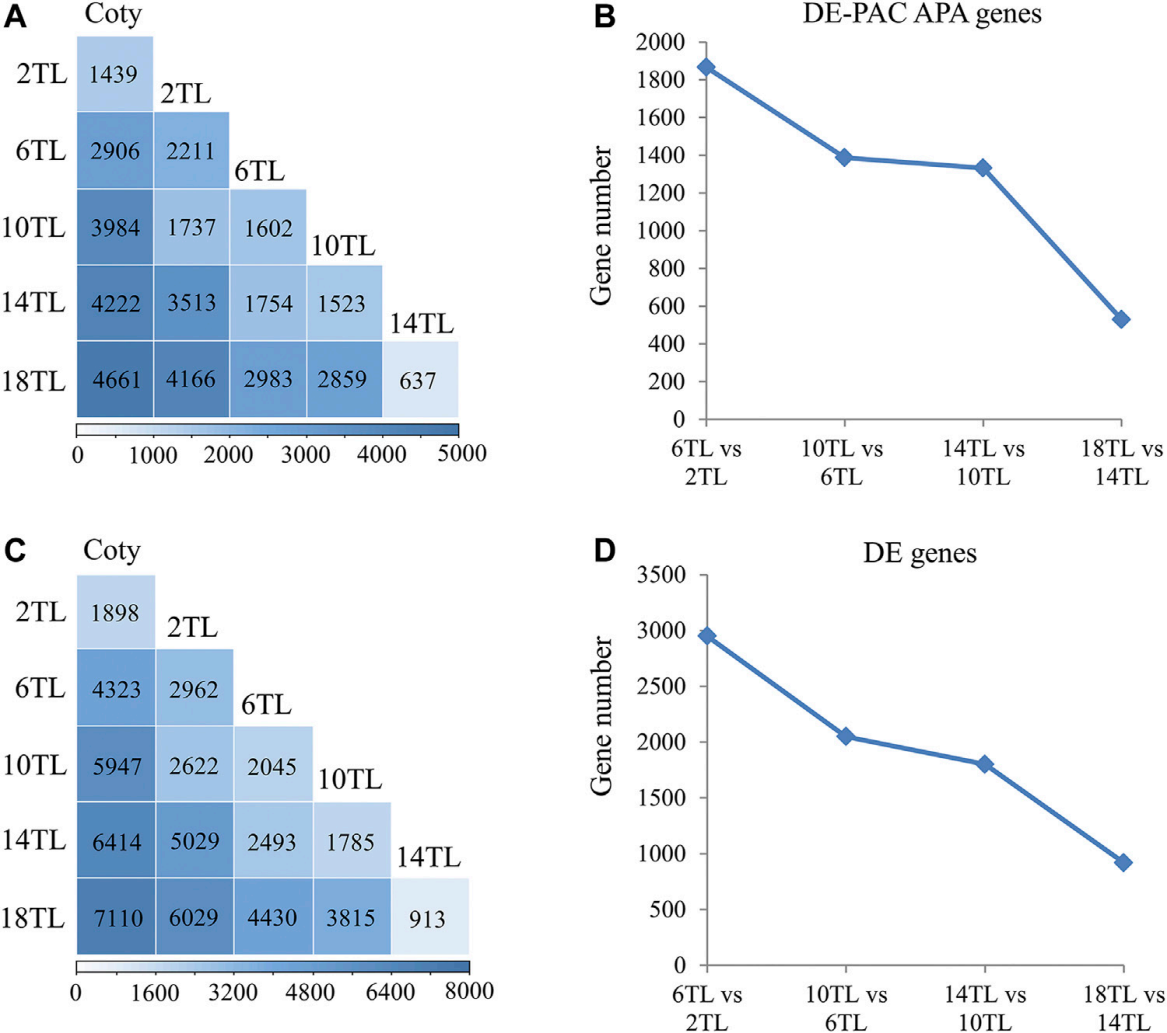
Analysis of differentially expressed poly(A) cluster (DE-PAC) APA genes and DE genes. Differential expression of PACs and genes were identified using the DESeq2 package. PAT read differences with *padj* < 0.05 were selected as DE genes and DE-PAC. **(A)** DE-PAC APA genes in pairwise comparisons. **(B)** The number of DE-PAC APA genes in adjacent true leaf comparison. **(C)** DE genes in pairwise comparisons. **(D)** The number of DE genes in adjacent true leaf comparison.

To further delve into the role of APA in leaf development, differentially utilized PAC APA genes were characterized using the DESeq2 package. DE-PACs tables were as shown in Supplementary Data 2–6. A large number of differentially expressed (DE) PAC APA genes were identified. Compared to cotyledon, over 1,000 DE-PAC APA genes were differentially utilized in 2 TL (Figure 3A). In the true leaf comparisons, the largest number of DE-PAC APA genes was found between 2 TL and 18TL, possibly because these true leaves were the farthest apart. Interestingly, the number of DE-PAC APA genes showed a reduction trend with adjacent leaf comparison (Figure 3B). DE genes appeared to show a similar tendency to DE-PAC APA genes (Figure 3C,D). These results indicated that APA might be involved in *Arabidopsis* leaf development.

**FIGURE 4:**
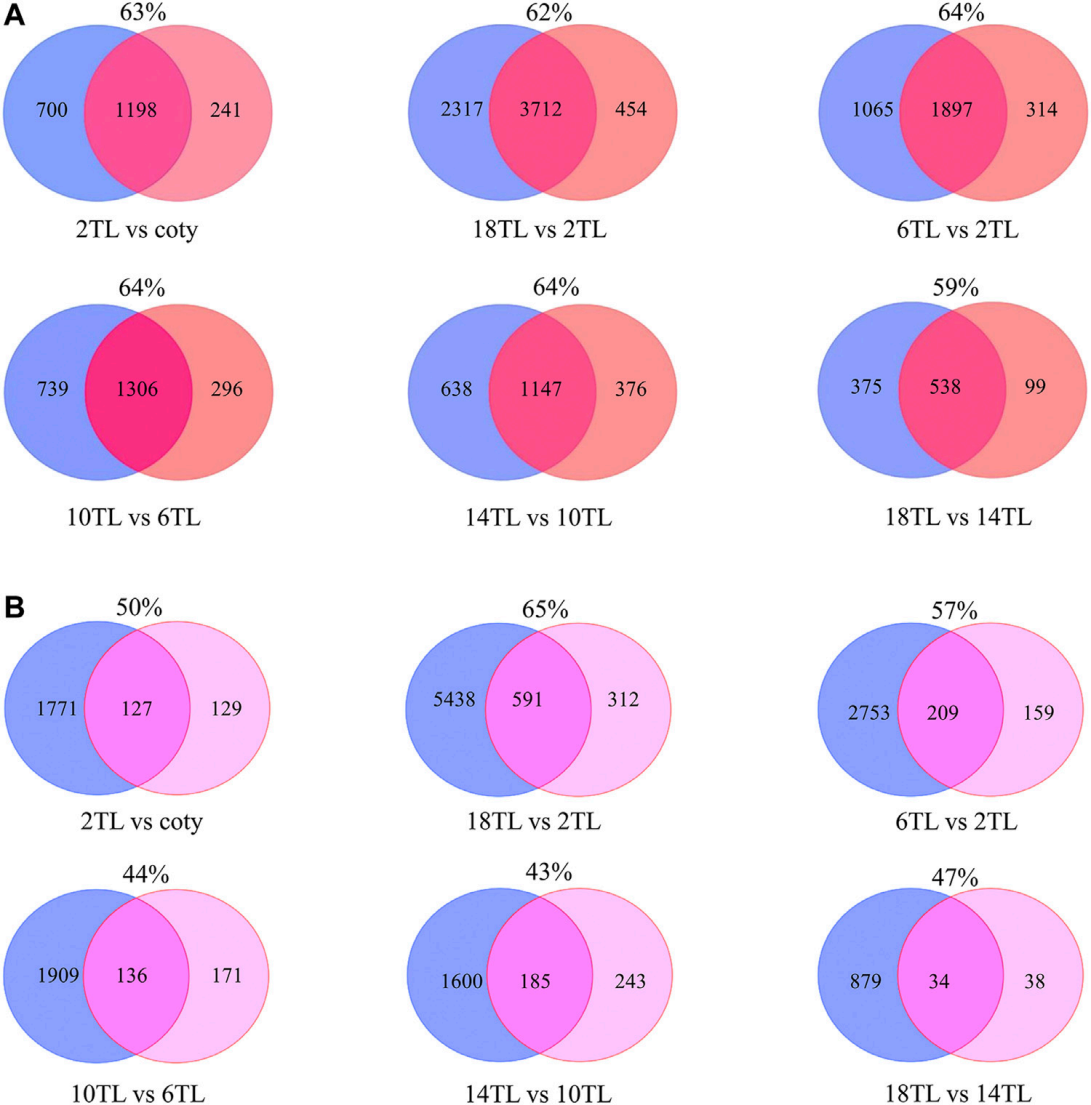
Venn diagram of DE-PAC APA genes and DE genes **(A)** and DE genes versus switch genes **(B)**. The blue circle represents DE genes; the orange circle represents DE-PAC APA genes; and the pink one represents switch genes. The number in the circles shows gene counts. The percentages represent the ratio of DE genes belonging to DE-PAC APA genes or the ratio of switch genes belonging to DE genes.

To assess the possible impact of APA on the expression of their associated genes, overlap analyses of DE-PAC APA genes and DE genes were explored (Figure 4). A large proportion of overlap (mostly >60%) of DE genes belong to DE-PAC APA genes in the adjacent leaf comparison and between 2 TL and 18 TL (Figure 4A). These results suggest that APA might play a role in modulating the gene expression, particularly in early- and late-developing leaves.

In order to exclude the effect of transcription on transcript level, poly(A) site usage (PAU) was applied to calculate switch genes. We found that over 200 genes switched APA site usage during leaf development (Figure 4B), except the pair of 18 TL and 14 TL comparisons that has only 72 switch genes which could be because they are both the newest leaves. In addition, over 40% switch genes altered their expression. More interestingly, over 50% of them regulated the transcript shifts and did not change the level of gene expression. This result further indicated that APA might mediate the gene expression involved in leaf development, regardless of the steady-state transcript level.

### Specific APA Genes Associated With Metabolic Processes and the Response of Hormones

**FIGURE 5:**
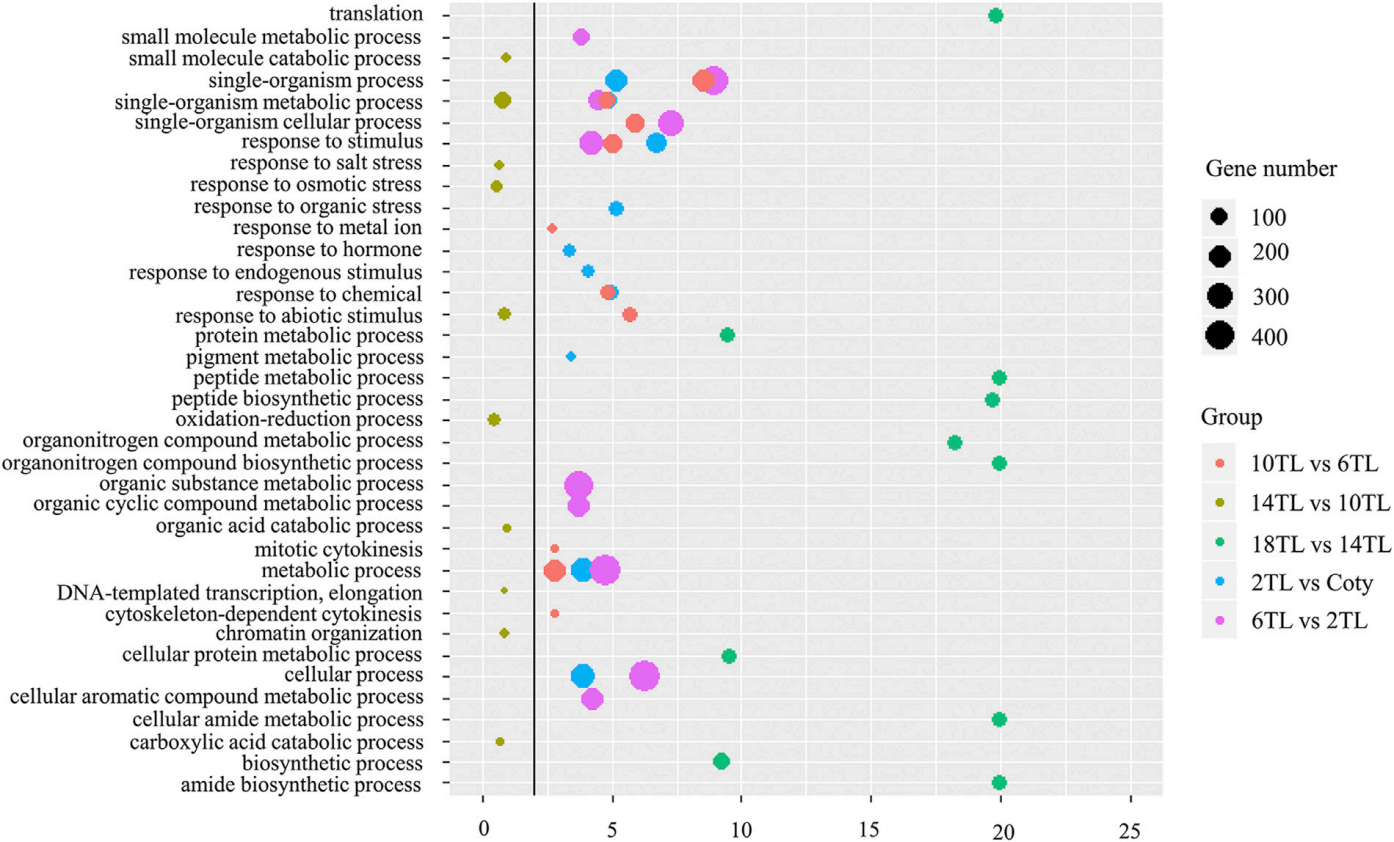
GO enrichment analyses of specific DE-PAC APA genes. GO analyses were carried out using agriGO (v.2.0). The top 10 GO terms of the biological process are shown. Solid line: FDR = 0.01. The colors represent different adjacent leaf comparisons, and the sizes of the circles represent gene numbers.

**FIGURE 6:**
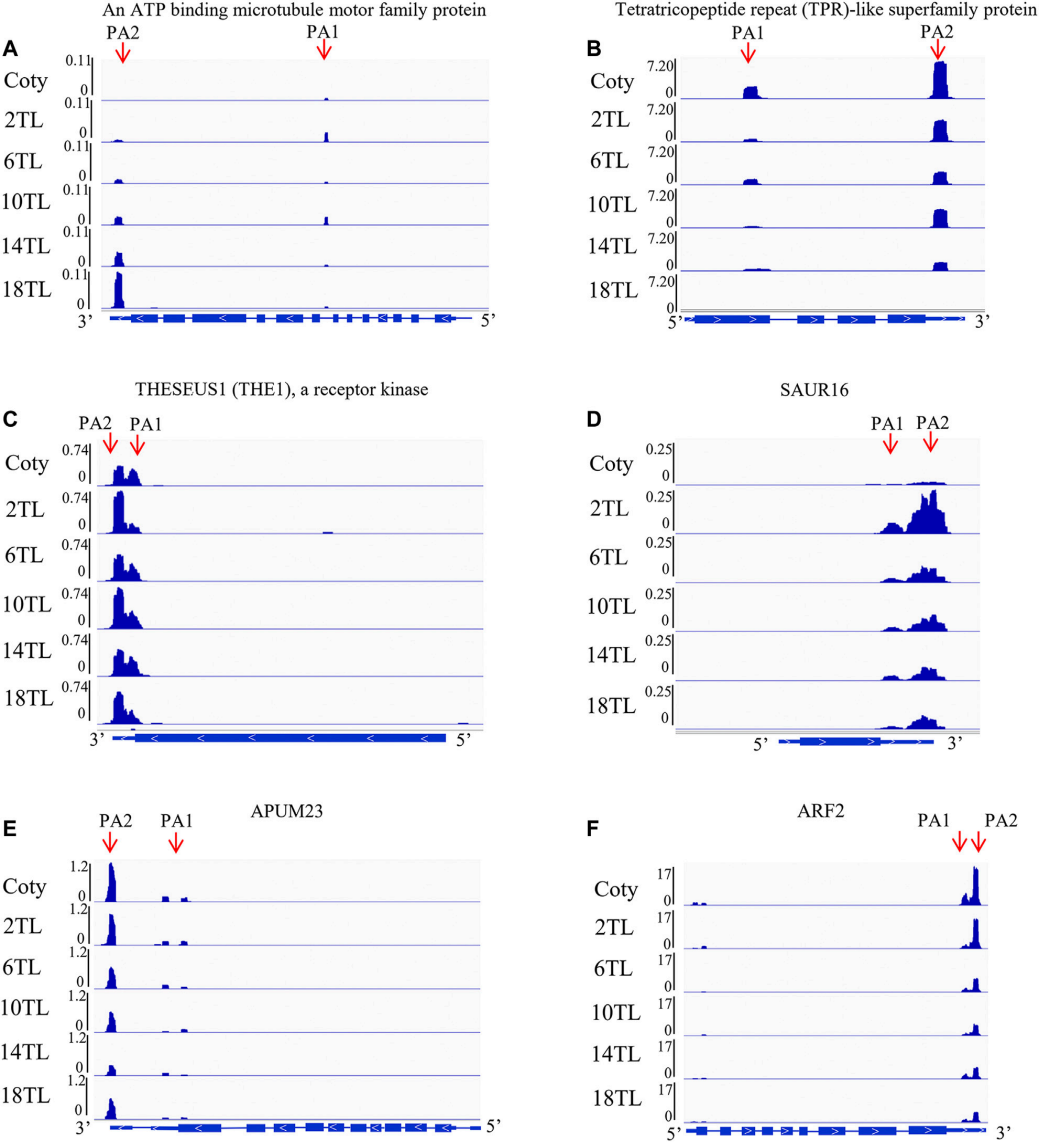
Sequencing coverage of genes visualized using integrative genomics viewer software. For the gene models on the bottom of each panel, the boxes denote exons, thin boxes denote untranslated regions, lines denote introns, and arrows denote transcription directions. PA indicates alternative polyadenylation site. **(A)** An ATP-binding microtubule motor family protein (AT4G38950). **(B)** tetratricopeptide repeat (TPR)-like superfamily protein (AT3G26580). **(C)** THESEUS1 (THE1) (AT5G54380), a receptor kinase. **(D)** SAUR16 (AT4G38860). **(E)** APUM23 (AT1G72320). **(F)** ARF2 (AT5G62000).

The abovementioned results showed that the expression of over 1,000 APA genes was altered. Overlap analyses were performed to reveal the specific DE-PAC APA genes among adjacent leaf comparisons. A total of 34 DE-PAC APA genes were found in all comparison groups, termed as common genes (Supplementary Figure S4). Interestingly, we also found hundreds of specific APA genes (Supplementary Figure S4). Gene ontology (GO) analyses were performed to explore the functions of specific APA genes. The top 10 GO terms for each pairwise comparison are represented in Figure 5. These include metabolic processes, cellular processes, and response to the stimulus. We found an ATP-binding microtubule motor family protein, which is related to the leaf size (Fleury et al., 2007). Distal transcript (PA2) abundance has an increased tendency with a new leaf growing up, but no expression in cotyledon (Figure 6A). Conversely, another tetratricopeptide repeat (TPR)-like superfamily protein, involving pathogen response and cell cycle (Ascencio-Ibanez et al., 2008), showed a proximal transcript (PA1) expression level that decreased with leaf development (Figure 6B). However, this gene was not expressed in the 18th leaf since it was not found in the PAT-seq data of this leaf stage.

Apart from the common GO terms, some specific GO terms were also detected. The numbers of specific GO terms were 4, 4, 3, 8, and 10 among 2 TL vs. cotyledon, 6 TL vs. 2TL, 10 TL vs. 6TL, 14 TL vs. 10TL, and 18 TL vs. 14 TL pairwise comparisons, respectively, suggesting that DE-PAC APA genes possess diversified functions at different leaf development stages. Notably, one of the specific GO terms among 2 TL vs. cotyledon was related to the response of hormones (Figure 5). Under this term, the expression of *THESEUS1* (*THE1*) encoding a receptor kinase, is regulated by BR and is required for cell elongation (Fujikura et al., 2014). Relative to the cotyledon, the distal transcript (PA2) abundance was greater in 2TL, while the proximal transcripts (PA1) were lower (Figure 6C). However, the expression of PA1 and PA2 was not obviously different among the true leaves. Another gene, small auxin upregulated RNA 16 (*SAUR16*), a *SAUR*-like auxin-responsive protein family, contributes to light-induced cotyledon opening (Dong et al., 2019). Notably, the PA1 and PA2 levels, particularly PA2, increased dramatically in the 2 TL relative to that of the cotyledon. In addition, the PA1 and PA2 abundance showed a downward trend during true leaf development (Figure 6D). While the expression level of *SAUR16* was in a dynamic mode, the differential APA could aid this change. Importantly, we found that the PA2 expression of Arabidopsis Pumilio (*APUM23*), functioning in leaf development (Huang et al., 2014), had a downward trend (Figure 6E). In addition, the PA1 abundance of auxin response factor 2 (*ARF2*) involving in leaf shape (Okushima et al., 2005), also showed a downward trend during leaf development (Figure 6F). These results demonstrated that APA assists to regulate the expression of key genes associated with leaf development.

### 3′UTR Tends to Shorten During True Leaf Development

**FIGURE 7:**
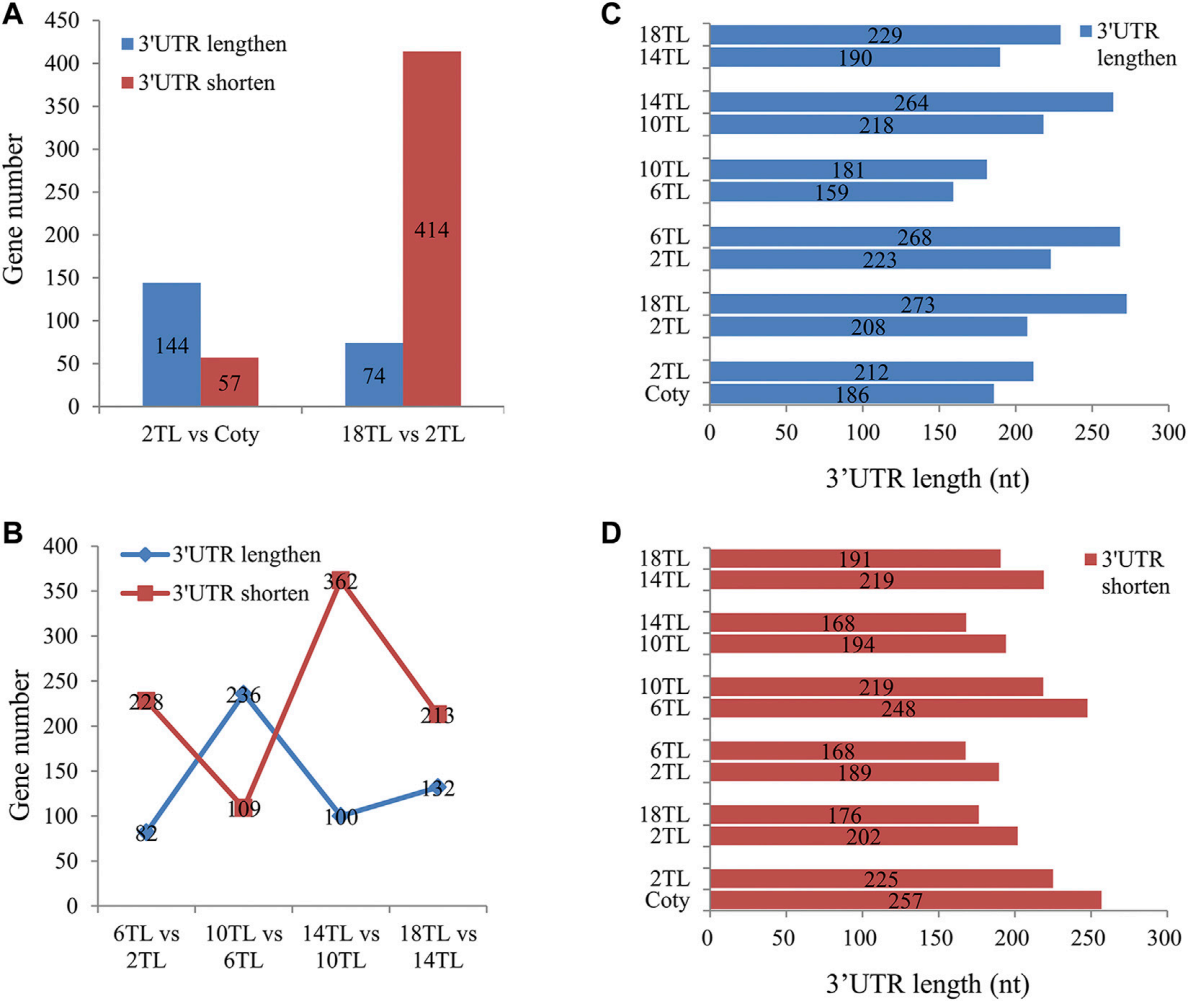
Analysis of 3′UTR length distributions. The *p value* < 0.01 was defined as the threshold for significance. **(A)** 3′UTR length changes between 2 TL and cotyledon, 2 TL and 18 TL. **(B)** 3′UTR length changes during true leaf development. **(C)** Median weighted length of 3′UTR in significantly lengthened genes in different comparison groups. **(D)** Median weighted length of 3′UTR in significantly shortened genes in different comparison groups.

As shown in Supplementary Figure S3, majority of the APA are located in 3′UTR, reflecting that 3′UTR APA may be more significant in regulating the gene expression. Thus, we focused on 3′UTR length analyses in different leaf comparisons. Interestingly, more genes used longer 3′UTR in 2 TL than cotyledon (Figure 7A), indicating a preference to use distal poly(A) sites in 3′UTR in 2 TL. However, compared to the 2TL, more genes used shorter 3′UTR in the 18TL, and the ratio of genes using proximal sites to those using distal ones is over five times (Figure 7A). Interestingly, during true leaf development, there is a tendency for more genes to use proximal sites in 3′UTR. Strangely, the 6 TL and 10 TL comparison groups showed an opposite trend (Figure 7B).

The median 3′UTR length of significantly lengthened and shortened genes were analyzed. In significantly lengthened genes of 3′UTR, the average 3′UTR length was 26 nucleotides longer in 2 TL than that in the cotyledon (Figure 7C). The most significant change in average 3′UTR length occurred between 2 TL and 18 TL, with an extension of 65 nucleotides. Similarly, in significantly shortened genes of 3′UTR, 32 and 26 nucleotides were shorter in 2 TL vs. cotyledon and 2 TL vs. 18 TL, respectively (Figure 7D). These results suggested that 3′UTR APA is associated with leaf development.

Previous studies demonstrated that shortening of 3′UTR in mammalian cells results in an increase in protein production, with the exception of miRNA-targeting genes (Sandberg et al., 2008). However, we found that there is no obvious correlation between the 3′UTR length switching and gene expression during leaf development, which is shown in Supplementary Figure S5 where the trend lines are all flat, indicative of no correlation.

## Discussion

The leaf is a basic organ that captures light energy and synthesizes organic compounds through photosynthesis. Leaves are the ultimate source of most food. Thus, an understanding of the underlying mechanism of leaf development will provide an insight into the basic processes in biology, and it may have a profound significance regarding strategies to improve the crop performance. Recent data indicate that some genes show a specific trend, either gradually increasing or decreasing during leaf development, indicating that leaf ontogeny is continuously changing, involving sequential and transient activation of regulators (Schmid et al., 2005; Efroni et al., 2008). However, the underlying molecular mechanism of leaf development remains to be elucidated.

Polyadenylation is an important co-transcription and mediates a set of biological processes (Liu et al., 2014). Previous research showed that APA of *Ethylene Response Factor4* (*ERF4*) is involved in the regulation of *Arabidopsis* leaf senescence (Riester et al., 2019). In our data, we found that the proximal transcript (PA1) of *ERF4* had a downward trend with a new leaf growing up (Supplementary Figure S6A). However, the distal transcript (PA2) had an anomaly change. In addition, *Hydroxypyruvate Reductase* (*HPR*), encoding a leaf-peroxisomal protein, has two transcripts by alternative splicing (Mano et al., 2000). Interestingly, we found that *HPR* contained different poly(A) sites, and the expression level of primary transcript (PA1) showed increased tendency during leaf development, suggesting that APA and alternative splicing may show a crosstalk relationship during leaf development.

In this study, we detected a dramatic change of genome-wide APA during *Arabidopsis* leaf development and found that the APA ratio, DE-PAC APA genes, and DE genes show a tendency to be reduced during leaf development, suggesting that APA might be involved in leaf development. In addition, APA is tightly associated with the expression of DE genes during leaf aging. Significant changes in 3′UTR APA occur during leaf development. Compared to cotyledon, the second true leaf prefers to use the distal poly(A) site in 3′UTR. However, an opposing trend is seen in terms of 3′UTR APA during true leaf development. Strangely, compared to 6TL, more genes in 10 TL showed a preference to use distal poly(A) site in 3′UTR, indicating a complex mechanism in addition to APA that is involved in leaf development.

Previous research showed that the deficiency of CFIm 68 and CFIm25 leads to a shift toward proximal poly(A) site usage in human HEK293 cells (Gruber et al., 2012; Martin et al., 2012). Interestingly, we also found that the expression level of CFIS1 and CFIS2, orthologous to CFIm 68 and CFIm25, respectively, had a downward trend during leaf development (Supplementary Figure S7A,B), indicating that the expression of CFIS1 and CFIS2 may contribute to the usage of poly(A) site in 3′UTR during leaf development. On the contrary, the loss of Pcf11 enhances distal poly(A) site usages in 3′UTR (Li et al., 2015). We also found that the expression of PCFS1 and PCFS5, the Arabidopsis orthologous of hPcf11 complex, showed an increased tendency during leaf development, except in 14 TL and 18 TL (Supplementary Figure S7A,B).

Previous studies have found that the length of 3′UTR modulates the expression level of genes in mammals (Sandberg et al., 2008). While there are about 40% of switch genes associated with the change of steady-state levels of their corresponding total transcripts, many other APA switch genes (>50%) did not show co-relationship with the steady-state level of transcripts. For the latter case, at the total transcript level, there is no obvious or little correlation between the length switching of 3′UTR (switch genes) and the steady-state gene expression levels (transcript copy numbers of genes) during leaf development (Supplementary Figure S5). This result points to a scenario where, in >50% of genes, different lengths of the APA switched transcripts may impact the translation efficiency, not the stability of these genes. Of course, this is a general trend line, not representing every gene, as many examples can be found in Supplementary Figure S5 where the data points are above or under the trend line. In fact, there are few miRNA targets that can be found between two APA sites in Arabidopsis (QQLi, unpublished observation) to justify the action of miRNA in mRNA degradations. On the other hand, what proportion of these hundreds of APA genes in fact participates in leaf development is an open question. It requires further studies to sort this out. In addition, our previous work also did not find an obvious negative correlation between 3′UTR length variation and gene expressions in different rice (*Oryza sativa*) tissues and different poly(A) factor mutants (Shen et al., 2008; Yu et al., 2019). Our results confirm that there may exist different mechanisms between what happened in plants and in mammals with respect to the correlation of 3′UTR length variation and the level of gene expression.

Leaf morphogenesis is an active process, controlled by plant hormones, transcriptional regulators, miRNAs, and mechanical forces, among factors (Wang et al., 2021). Significantly, we found that some key APA genes are related to leaf development. Interestingly, the distal transcript (PA2) abundance of an ATP-binding microtubule motor family protein, which is related to leaf size (Fleury et al., 2007), has an increased tendency toward growth with the appearance of a new leaf. The size and shape of a leaf are related to cell proliferation and differentiation. We also found that the proximal transcript (PA1) expression level of tetratricopeptide repeat (TPR)-like superfamily protein, involved in the cell cycle (Ascencio-Ibanez et al., 2008), decreased along with leaf development. In addition, the accumulating evidence demonstrated that plant hormones, including auxin, cytokinin, and BR have important roles during leaf development. GO enrichment analyses of the specific DE-PAC APA gene were carried out in this study. One of the terms, response to hormone, was found between the cotyledon and the 2 TL comparisons. SAUR16, a SAUR-like auxin-responsive protein family, contributes to light-Induced cotyledon opening (Dong et al., 2019). Notably, the PA1 and PA2 levels, particularly PA2, increased dramatically in the 2 TL relative to the levels in the cotyledon. In addition, PA1 and PA2 abundance show a downward trend during true leaf development. These results indicate that APA regulated the expression of key genes associated with leaf development. Although some genes have been identified as participating in leaf development in recent years, the molecular mechanism is not fully clear. With the development of genomic approaches, it will help to bring insights into mechanisms of the dramatic leaf development process.

## Data Availability

The datasets presented in this study can be found in online repositories. Raw high-throughput sequence data for this study were deposited in the National Center for Biotechnology Information SRA database under the code PRJNA778569.
