# Supplementary material for: Signatures of mRNA Alternative Polyadenylation in Arabidopsis Leaf Development
Source: Front Genet. 2022 Apr 26;13:863253. doi: 10.3389/fgene.2022.863253 (PMC9086830; doi:10.3389/fgene.2022.863253)
Supplement: Supplementary file 7 [file DataSheet1.PDF]

Yu et al Supplemental Figures

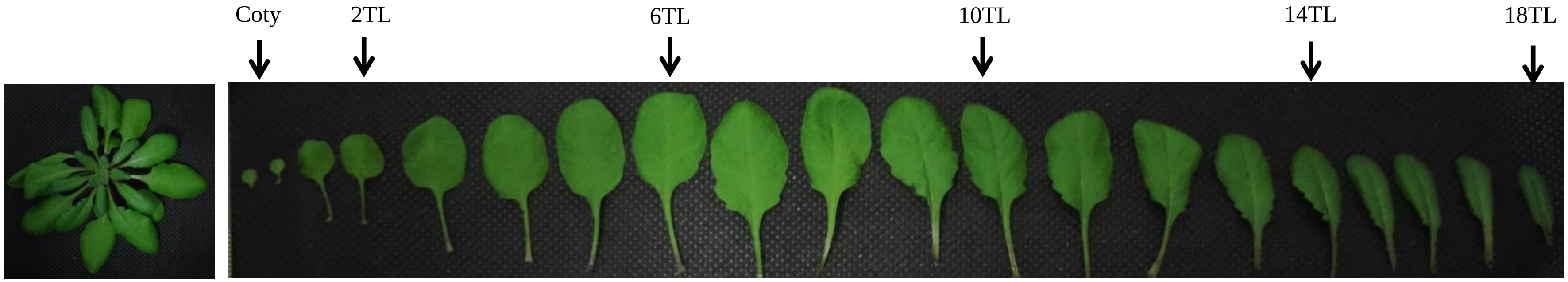

Supplementary Figure 1. Phenotype of leaves in 5-week-old plants under a 12:12 h light/dark cycle. A whole plant (left) and excised leaves are arranged according to their age from older (left) to younger (right). The arrow indicates the leaf sampled for transcriptome analyses in this study. Cotyledon, Coty; True leaf, TL; number before TL indicates the order of the leaves, e.g.2TL, the second true leaf.

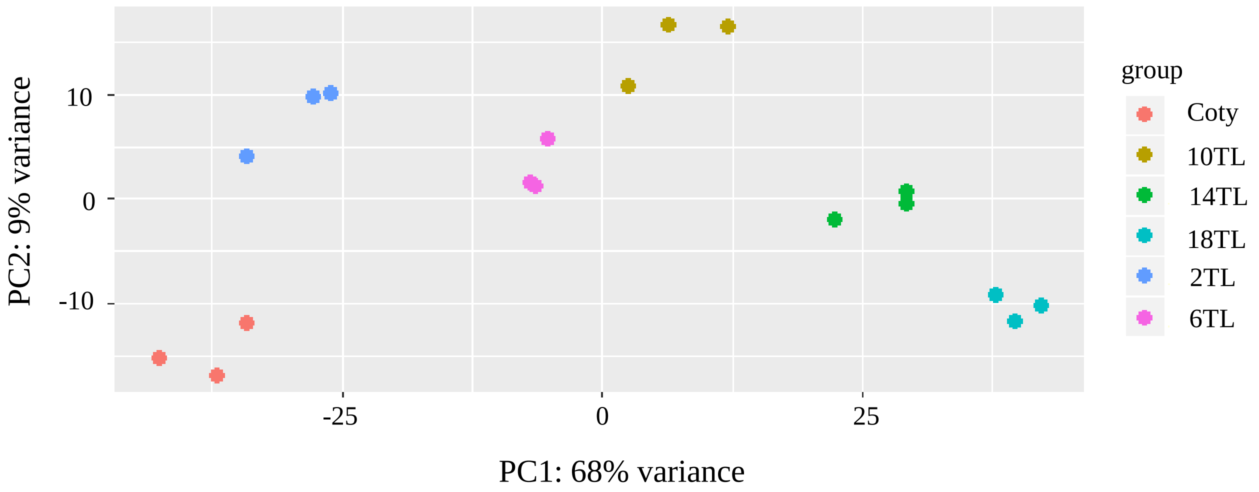

Supplementary Figure 2. Principal Component Analysis of repeatability of three biological replications. The 18 samples shown in the 2D plane spanned by their first two principal components based on the normalized expression values of each PAC. Each color represents a sample consisting of three repeats.

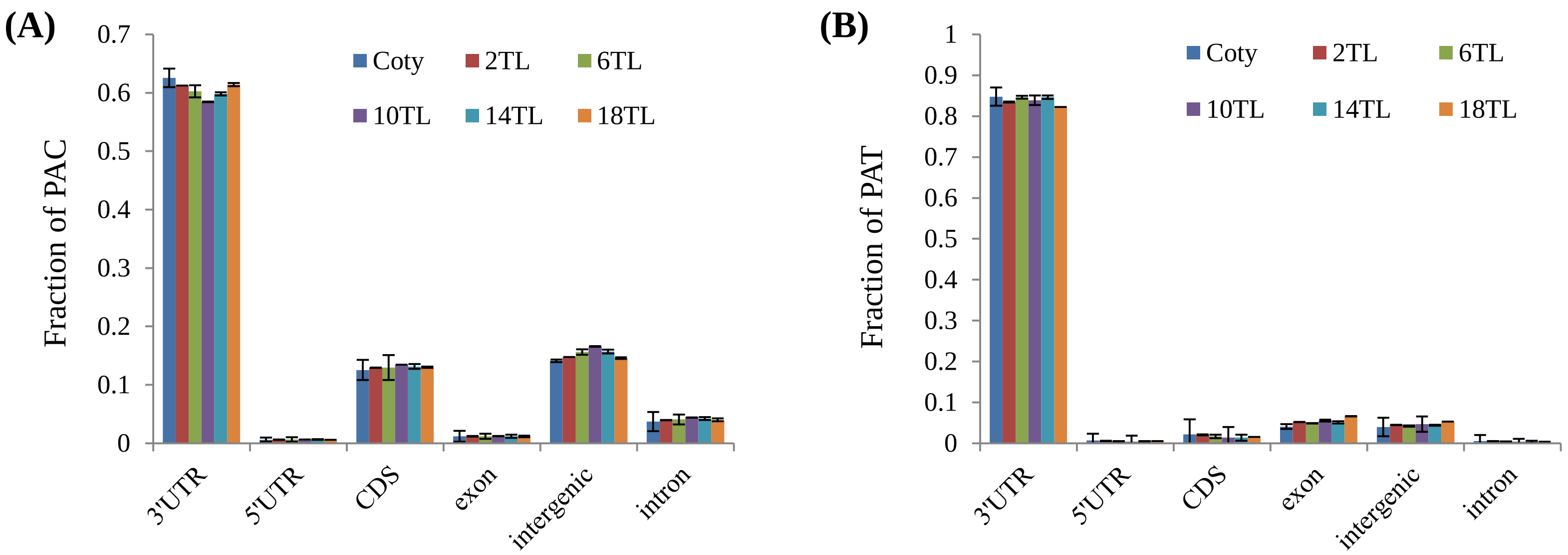

Supplementary Figure 3. The distribution of PACs and PATs in genic regions. (A), PACs distribution. **(B)**, PATs distribution. One-way ANOVA was used to analyze significant differences among adjacent leaf comparisons. The *p-value* of all adjacent leaf comparison was more than 0.05.

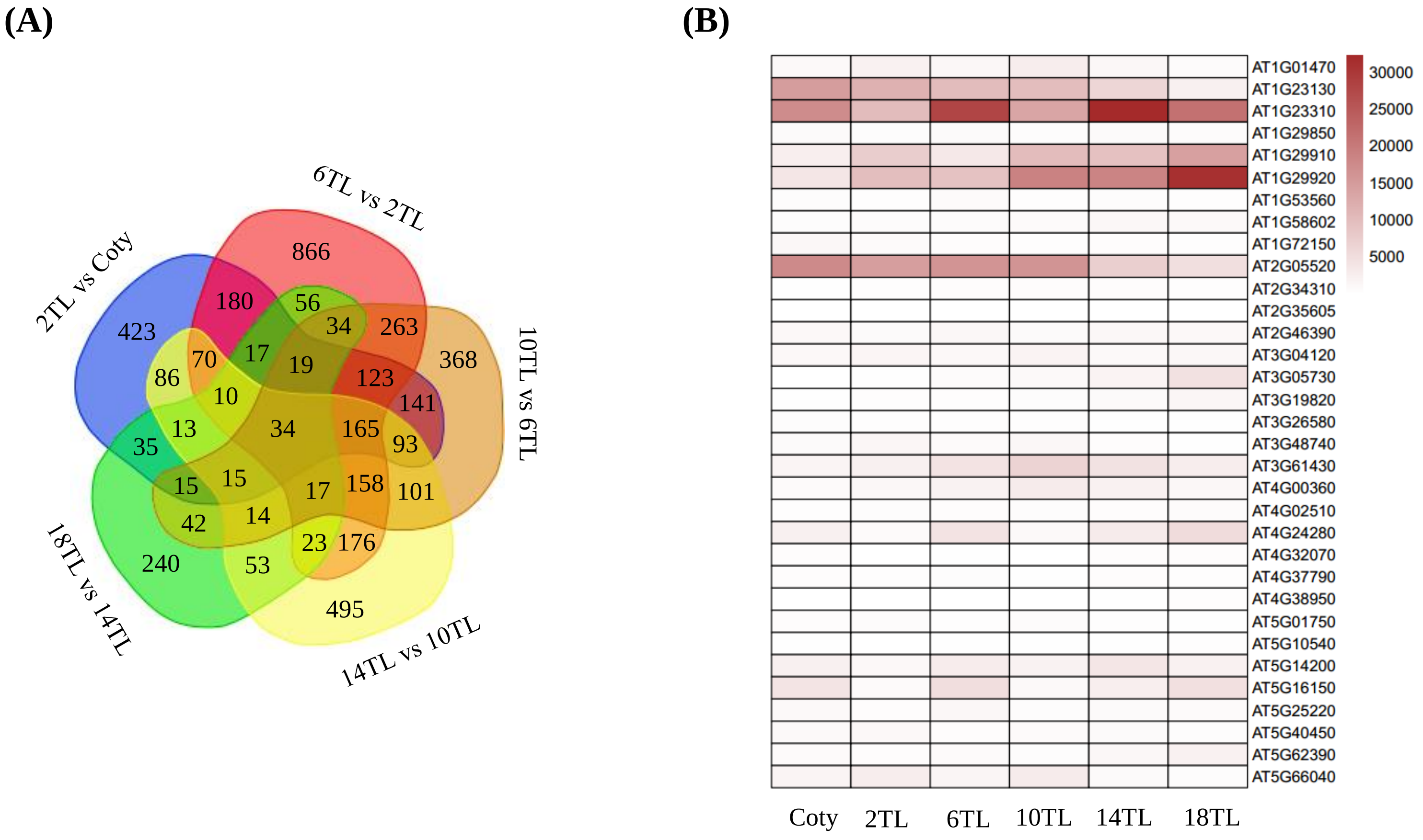

Supplementary Figure 4. Venn diagram of differentially expressed (DE) PAC APA genes (A) and heat map of common APA genes normalized data (B). PAT read differences with *padj*<0.05 were selected as DE-PAC among adjacent leaf comparisons. DESeq2 package was used to normalize the raw data.

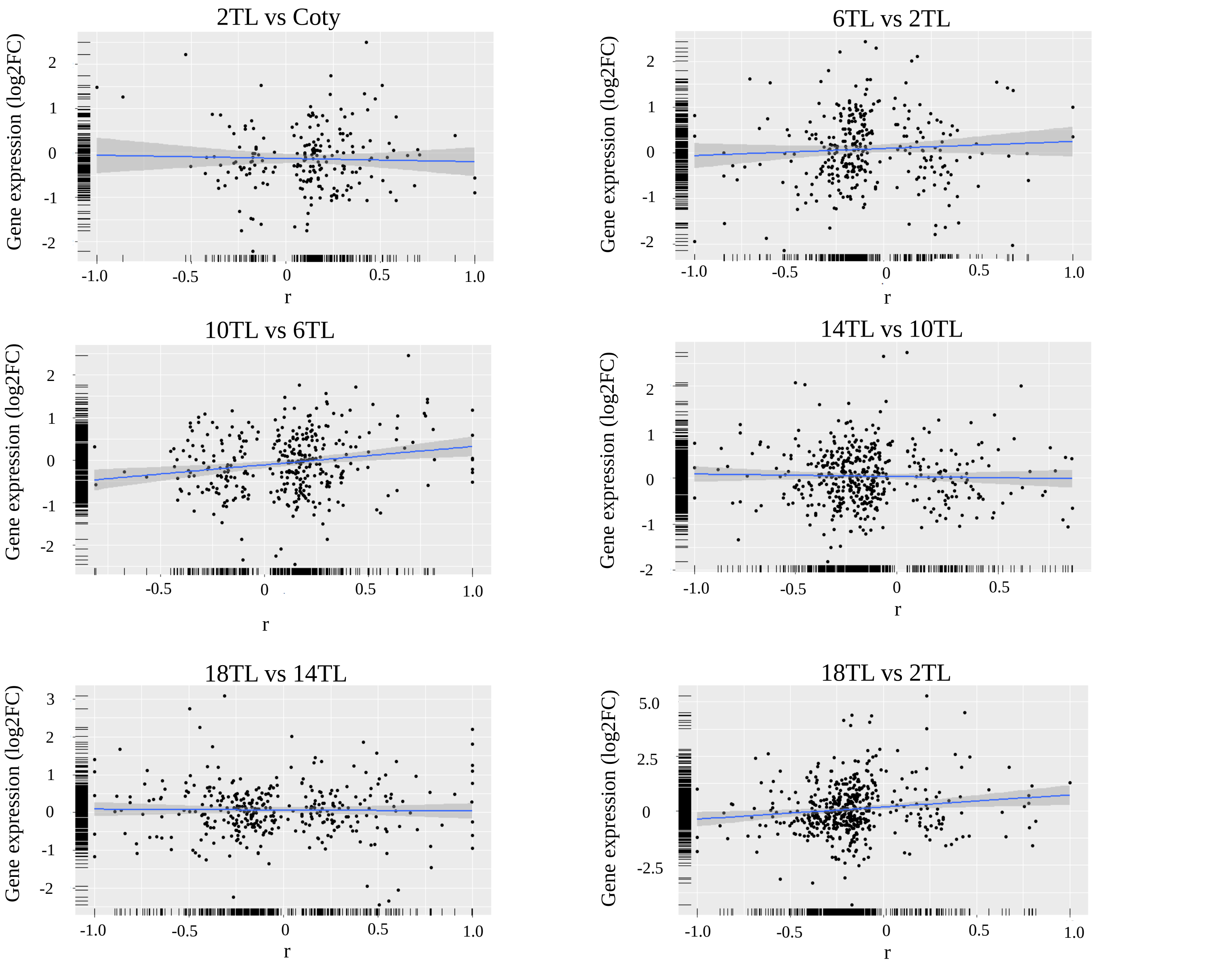

Supplementary Figure 5. The relationship between 3'UTR length and gene expression. Pearson product moment correlation coefficient is plotted against the log2 fold change between different samples. The *p-value*<0.01 was defined as 3'UTR length significant change. r<0 indicates 3' UTR shorten; Conversely, r>0 indicates 3' UTR lengthen.

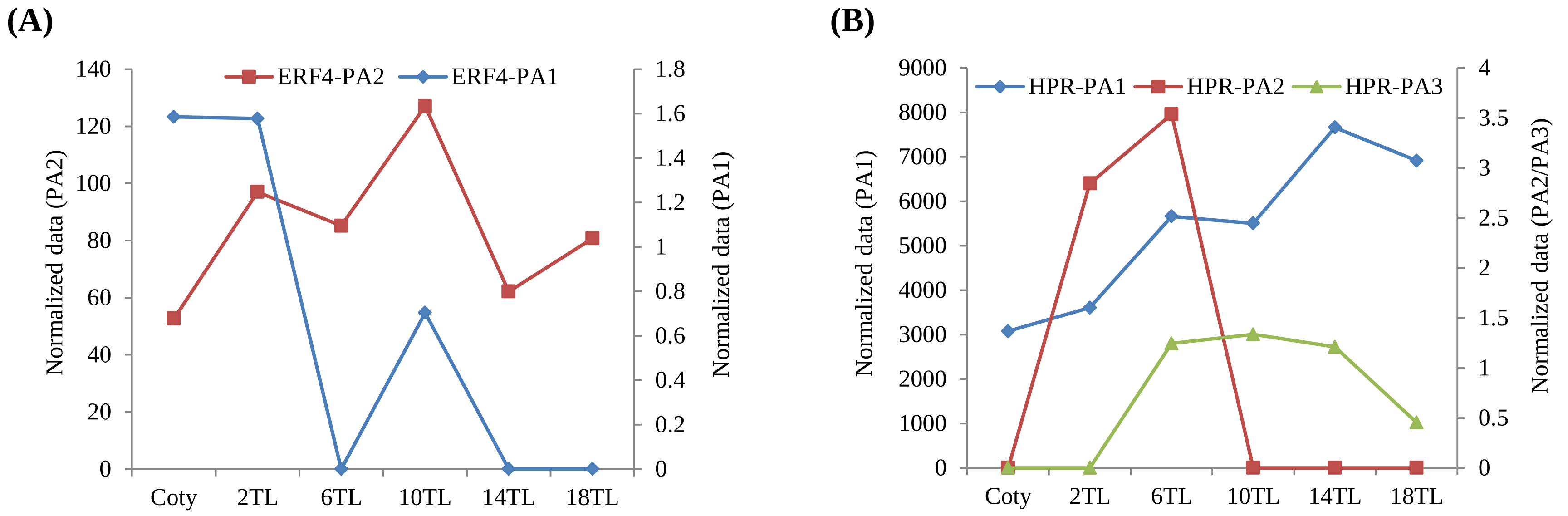

Supplementary Figure 6. Normalized data of ERF4 (A) and HPR (B) transcripts. DESeq2 package was used to normalize the raw data. PA indicates alternative polyadenylation sites.

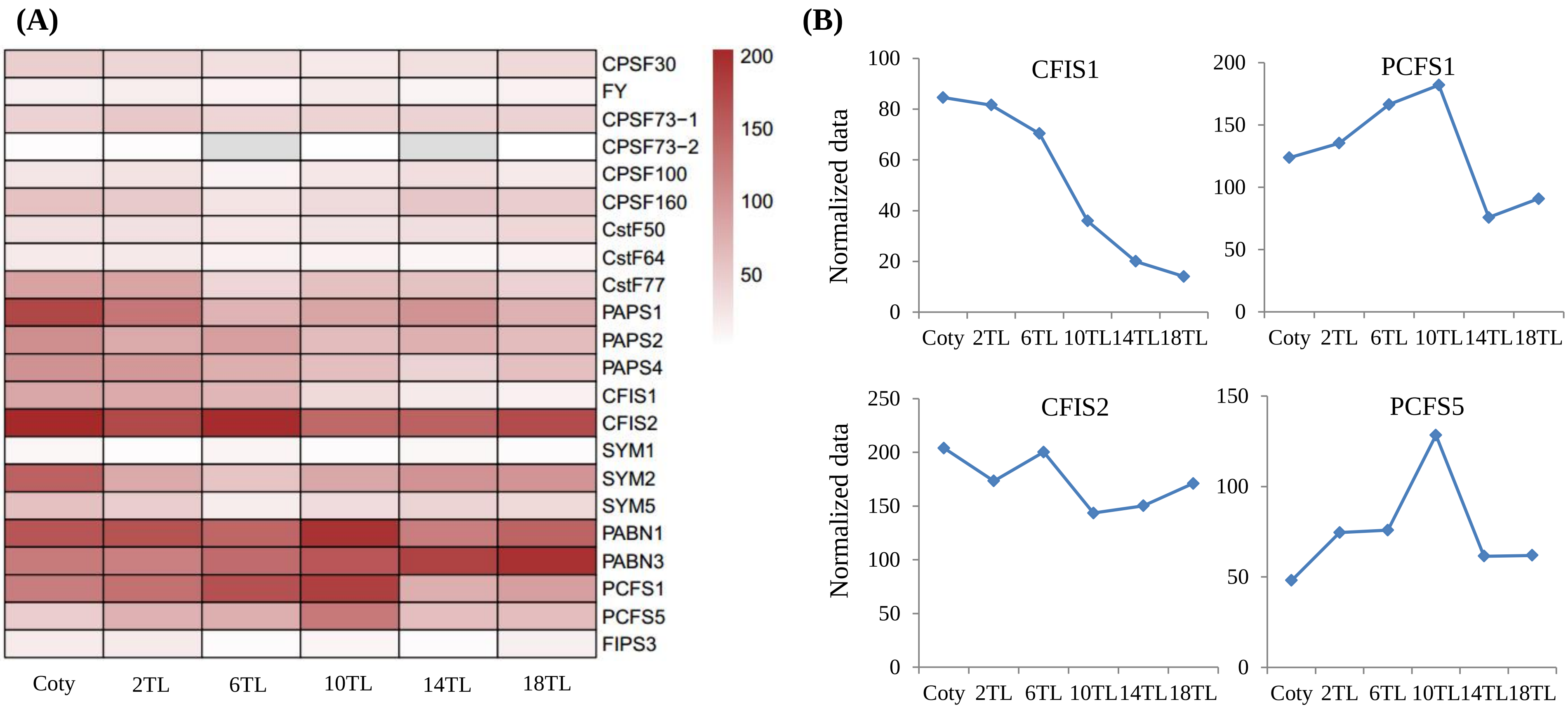

Supplementary Figure 7. Normalized data of poly(A) factors. (A), Heat-map shows poly(A) factors expression level. **(B)**, The expression level of CFIS1 and CFIS2, rthologous to CFIm 68 and CFIm25, and the expression of PCFS1 and PCFS5, the *Arabidopsis* orthologous of hPcf11 complex. DESeq2 package was used to normalize the raw data.
